# Supplementary material for: Pervasive structural heterogeneity rewires glioblastoma chromosomes to sustain patient-specific transcriptional programs
Source: Nat Commun. 2024 May 9;15:3905. doi: 10.1038/s41467-024-48053-2 (PMC11082206; doi:10.1038/s41467-024-48053-2)
Supplement: Supplementary file 1 — Supplementary Information [file 41467_2024_48053_MOESM1_ESM.pdf]

SUPPLEMENTARY INFORMATION

Pervasive structural heterogeneity rewires glioblastoma chromosomes to sustain patient-specific transcriptional programs

Ting Xie[1], Adi Danieli-Mackay[1], Mariachiara Buccarelli[2], Mariano Barbieri[1], Ioanna Papadionysiou[1], Q. Giorgio D'Alessandris[3,4], Claudia Robens[5], Nadine Übelmesser[1], Omkar Suhas Vinchure[6], Liverana Lauretti[3], Giorgio Fotia[7], Roland F. Schwarz[5,8], Xiaotao Wang[9,10], Lucia Ricci-Vitiani[2], Jay Gopalakrishnan[6,11], Roberto Pallini[3,]* & Argyris Papantonis[1,]*

[1] *Institute of Pathology, University Medical Center Göttingen, Göttingen, Germany*

[2] *Department of Oncology and Molecular Medicine, Istituto Superiore di Sanità, Rome, Italy*

[3] *Department of Neuroscience, Catholic University School of Medicine, 00168 Rome, Italy*

[4] *Department of Neuroscience, Fondazione Policlinico Universitario A. Gemelli IRCCS, Roma, Italy*

[5] *Institute for Computational Cancer Biology (ICCB), Center for Integrated Oncology (CIO), Cancer Research Center Cologne Essen (CCCE), University of Cologne, Cologne, Germany*

[6] *Institute of Human Genetics, University Hospital and Heinrich-Heine-University Düsseldorf, Düsseldorf, Germany*

[7] *Centre for Advanced Studies, Research and Development in Sardinia (CRS4), Pula, Italy*

[8] *Berlin Institute for the Foundations of Learning and Data (BIFOLD), Berlin, Germany*

[9] *Institute of Reproduction and Development, Fudan University, Shanghai, China*

[10] *Research Units of Embryo Original Diseases, Chinese Academy of Medical Sciences, Shanghai, China*

[11] *Institute of Human Genetics, Jena University Hospital and Friedrich Schiller University of Jena, Jena, Germany*

* Correspondence: A.P., argyris.papantonis@med.uni-goettingen.de
R.P., roberto.pallini@unicatt.it

This document contains **Supplementary Figures 1-10** and **Supplementary Tables 1-2**.

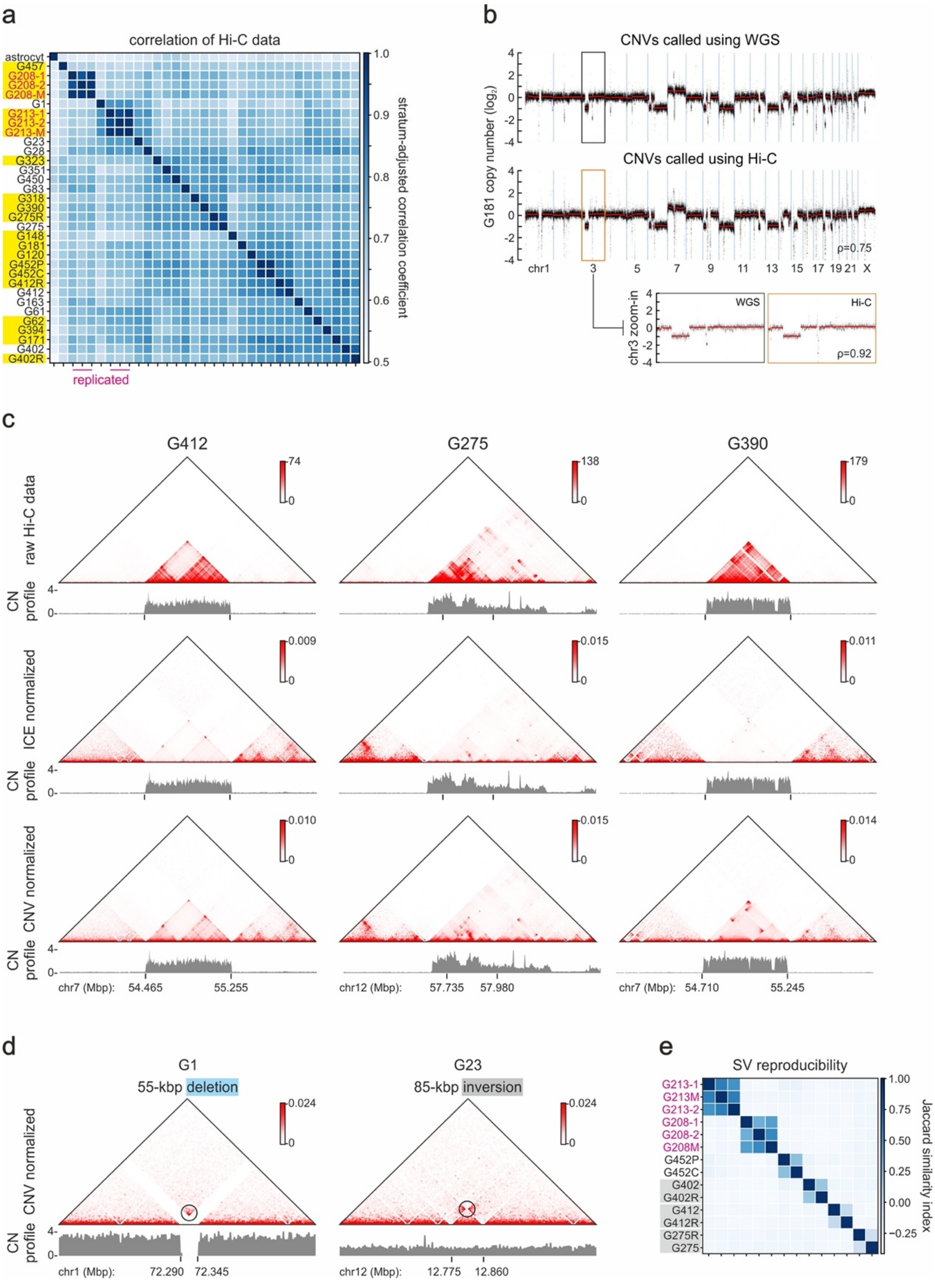

Supplementary Fig. 1. Evaluation of Hi-C reproducibility, CNV segmentation, and normalization. a, Heatmap showing stratum-adjusted correlation between 10 kbp-resolution raw Hi-C contact matrices from all 28 GSC lines. Biological replicates from the same line (-1/-2) and their merged map (M) are indicated. Data from relapse GSCs are highlighted (yellow). **b,** Comparison of whole-genome CNV computation using G181 WGS (via *CNVkit*) or Hi-C data (via

NeoLoopFinder). A zoom-in for the CNVs identified along chr3 is provided. Pearson's correlation coefficients (ρ) are shown for both comparisons. **c,** Comparison of 5 kbp-resolution raw (top), ICE-normalized (middle) or CNV-normalized Hi-C contact matrices (bottom) around exemplary amplified regions (CNV profiles aligned below). **d,** Exemplary 5-kbp resolution Hi-C contact maps showing signal characteristic of short-range SVs for a deletion in G1 (left) and an inversion in G23 (right). **e,** Heatmap showing similarity of SVs discovered in Hi-C data of 12 exemplary GSCs. Biological replicates from the same line (-1/-2) and their merged map (M) are indicated.

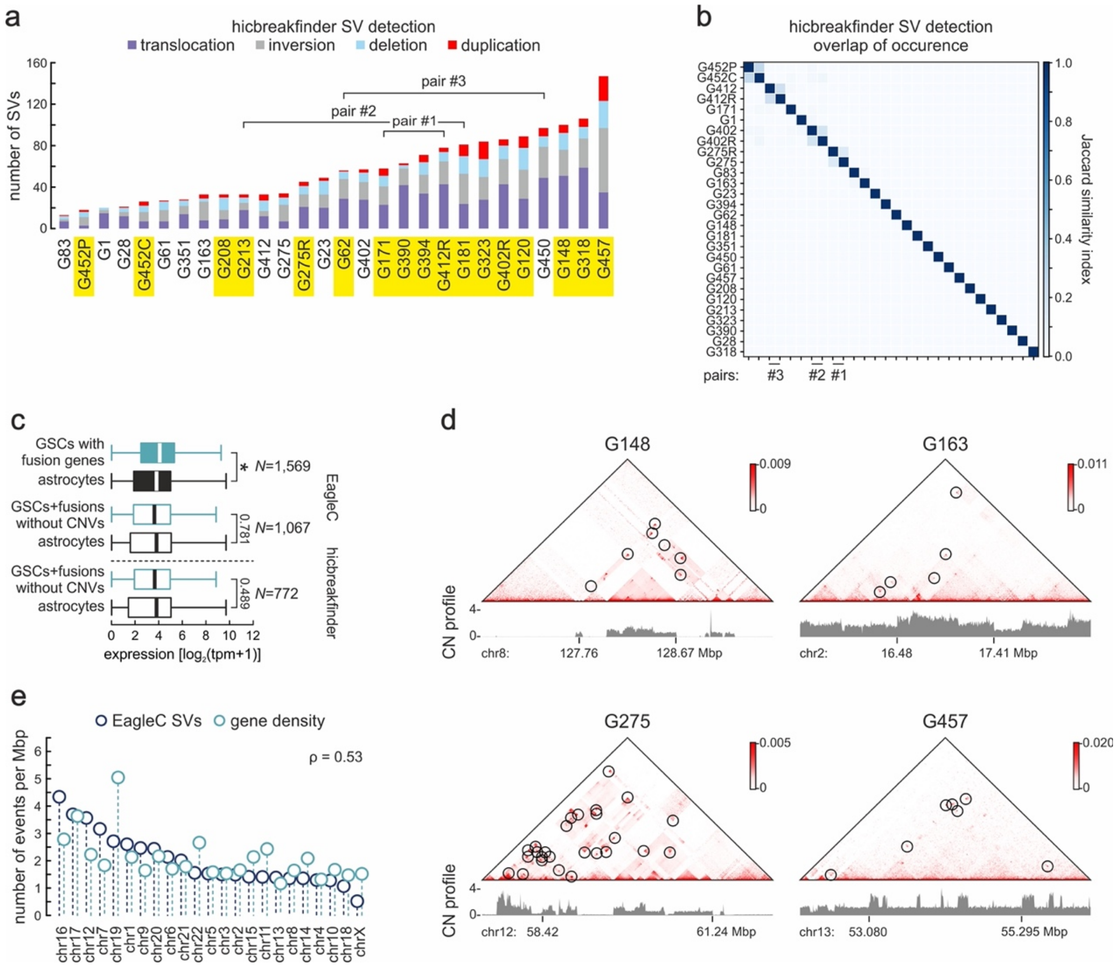

Supplementary Fig. 2. Clustered SV occurrence and gene fusions along GSC chromosomes. **a,** Bar plot showing the number of SV types identified using *hicbreakfinder* in each GSC line. Lines from relapse tumors are highlighted (yellow). **b,** Jaccard similarity index of the SVs described in panel a. **c,** Box plots (bands show the mean, each box extends between 1st and 3rd quartile, and whiskers extend 1.5x the interquartile range) showing expression levels of 1,569 gene fusions identified on the basis of EagleC SVs and of 772 identified on the basis of *hicbreakfinder*-deduced SVs in GSCs before (green) and after filtering out CNV-associated fusions (of >1.5 CNV) compared to their individual counterparts in astrocytes (black). **P* = 1.072e-08, two-sided Wilcoxon rank-sum test. Source data for this panel are provided as a Source Data file. **d,** Exemplary Hi-C contact maps from 4 GSC lines showing SV clustering in 3-Mbp stretches of different chromosomes. Source data for this panel are provided as a Source Data file. **e,** Lollipop plots showing the number of EagleC-deduced SVs (dark blue) or of genes per Mbp of each chromosome (light blue). The Pearson's correlation coefficient (ρ) for the two datasets is calculated.

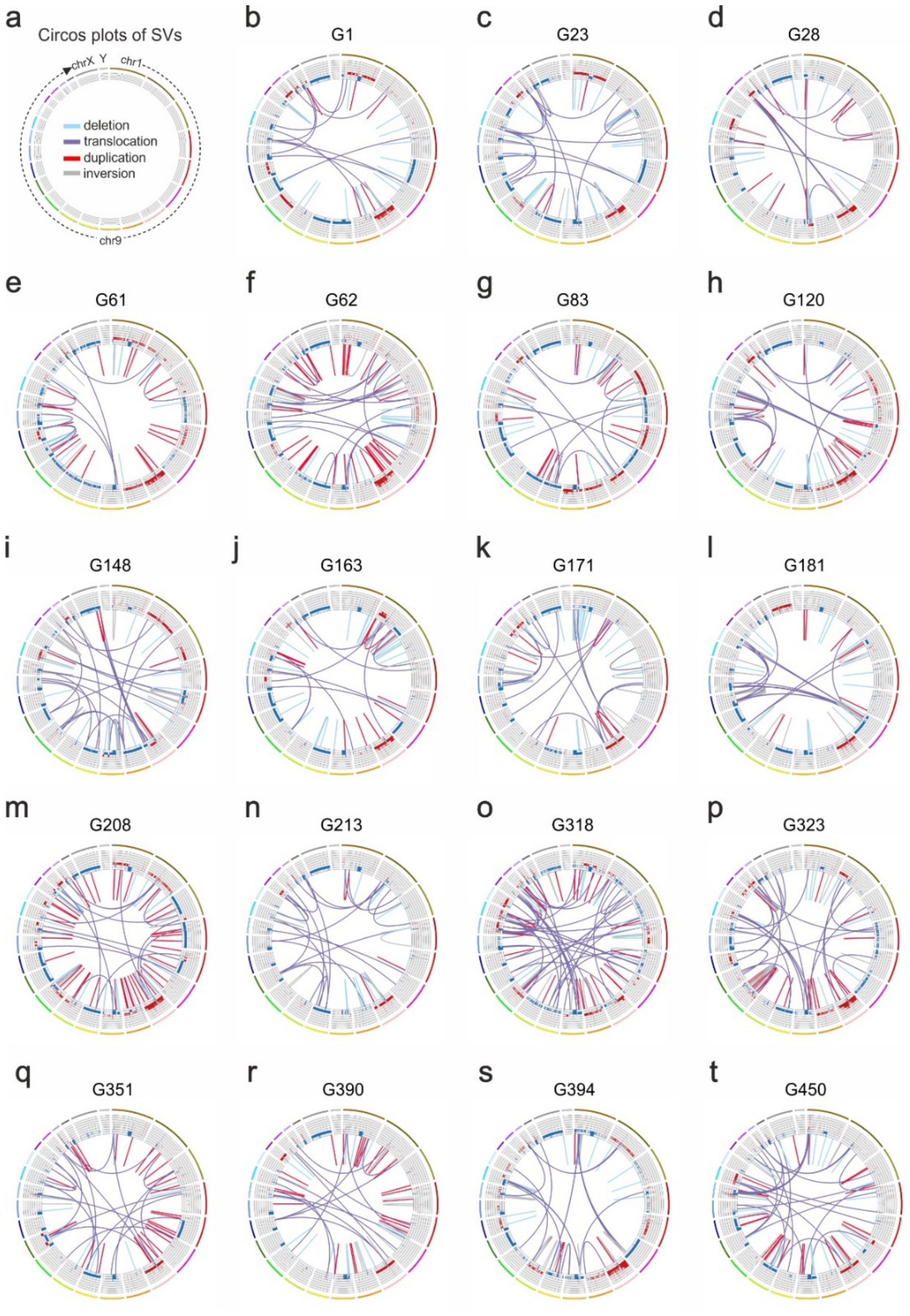

Supplementary Fig. 3. Distribution of SVs across GSC lines. a, Key showing the positions of chromosomes (outer tracks) and the color code for SVs in Circos plots (inversions – grey; deletions – light blue; duplications – red; translocations – purple). **b-t,** Circos plots of EagleC SVs and CNVs detected in 19 GSC Hi-C datasets. Inner tracks: gain (red, >2 copies) or loss of genomic segments (blue, <2 copies); lines: SVs are color-coded as in panel a.

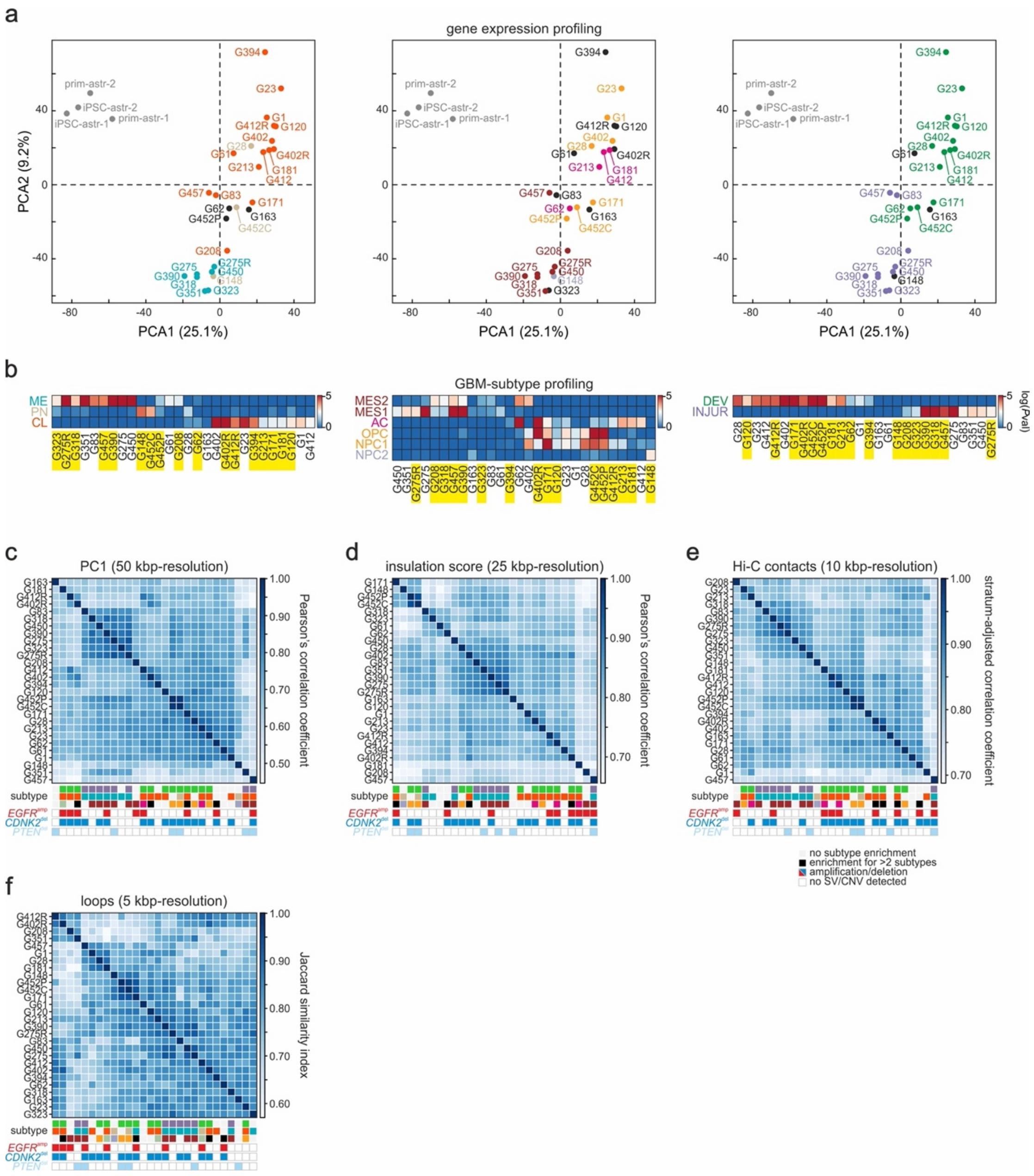

Supplementary Fig. 4. Discriminating GSC subtypes based on 3D genome organization features. a, Left: PCA plot of RNA-seq replicates from 28 GSC lines classified as mesenchymal (green), classical (orange) or proneural (brown). Middle: Same PCA plot, but with the 28 GSCs classified as mesenchymal (dark red), astrocyte-like (magenta), OPC-like (yellow) or neural precursor-like (grey). Right: Same PCA plot, but with the 28 GSCs classified as developmental (green) or injury response -like (purple). In all cases, data from primary or iPSC-derived astrocytes provide a control. **b,** Expression-based subtyping of all GSCs into the three classifications from panel a, scored by empirically-derived *P*-values for each signature. GSCs derived from relapse tumors are indicated (yellow highlight). **c,** Heatmap showing unsupervised clustering and Hi-C correlation on the basis of PC1 values called at 50-kbp resolution data for all GSC lines. The color code (below) reflects the subtype of each line according to panel b, as well as the status of driver mutations in *EGFR* amplification, and *CDKN2A/B* or *PTEN* deletion.

d, As in panel c, but based on insulation scores calculated from 25 kbp-resolution Hi-C data. **e,** As in panel c, but computing SCC correlation for all Hi-C contacts at 10-kbp resolution. **f,** As in panel c, but computing the Jaccard similarity index for loop overlap between GSCs. Two loops are considered “the same” if the midpoint of each anchor in one loop is within <50 kbp from the anchor midpoint in the other.

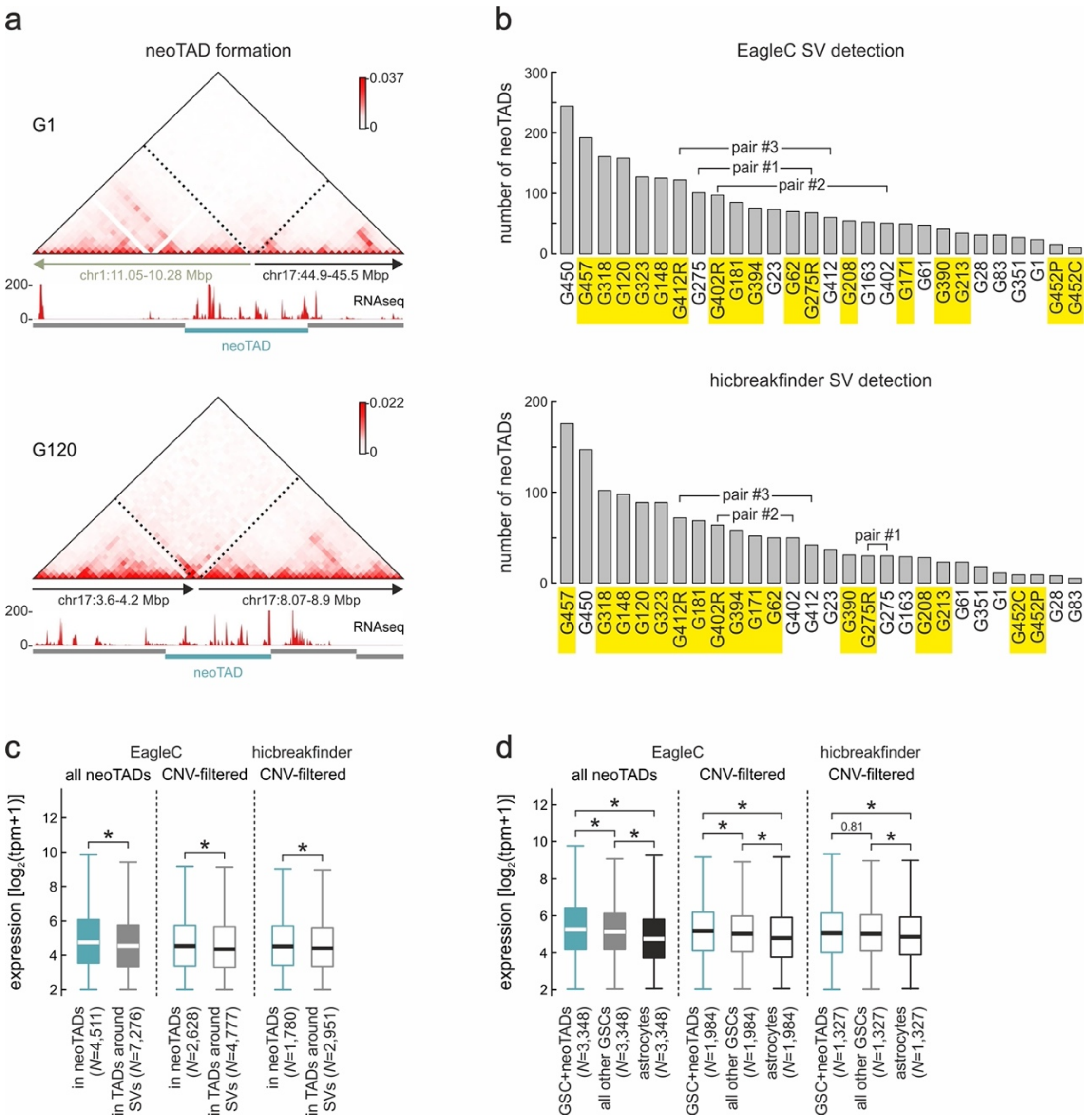

Supplementary Fig. 5. SVs give rise to GSC-specific neoTADs. a, Exemplary Hi-C contact maps around a G1 translocation (top) and a G120 deletion (bottom) giving rise to neoTADs (green rectangle). **b,** Bar plots showing the number of neoTADs identified in each GSC line using EagleC (top) or *hicbreakfinder* (bottom). GSCs from relapse tumors are indicated (yellow). **c,** Box plots (bands show the mean, each box extends between 1st and 3rd quartile, and whiskers extend 1.5x the interquartile range) show mean expression of genes in neoTADs (green) identified using EagleC or *hicbreakfinder* compared to genes in neighboring TADs (grey) before and after filtering CNV-associated genes (of >1.5 CNV). **P*= (left to right) 0.0004, 0.0401 and 0.021, two-sided Mann-Whitney U-test. Source data for this panel are provided as a Source Data file. **d,** As in panel d, but for genes in GSC-specific neoTADs (green) identified using EagleC or *hicbreakfinder* compared to the same genes in GSCs without neoTADs (grey) or in astrocytes (black) before and after filtering out CNV-associated genes (with >1.5 CNV). * *P*= (left to right) 4.83e-85, 3.44e-19 and 5.73e-76 (for all neoTADs), 2.00e-11, 2.08e-03 and 2.19e-24 (for CNV-filtered neoTADs), and 4.49e-08 and 2.24e-13 (for CNV-filtered neoTADs from *hicbreakfinder*), respectively; two-sided Wilcoxon rank-sum test. Source data for this panel are provided as a Source Data file.

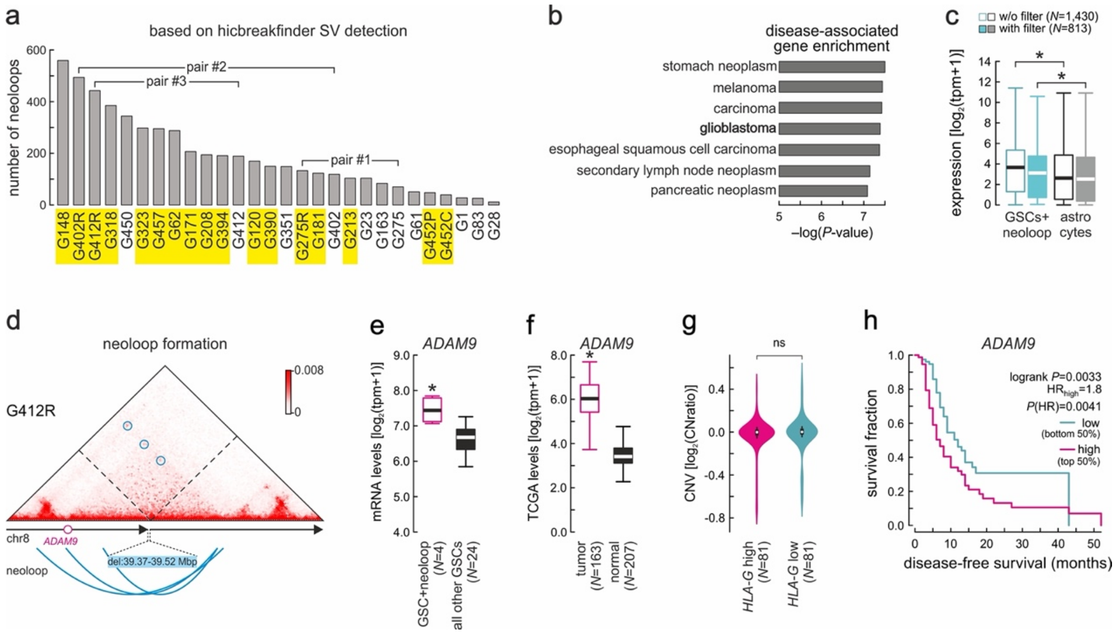

Supplementary Fig. 6. Neoloops are pervasive and associated with poorer prognosis. a, Bar plot showing the number of neoloops identified using *hicbreakfinder* in each GSC line. Lines derived from relapse tumors are indicated (yellow). **b,** Signatures of neoloop-associated genes from panel a and the DisGeNET database (*P*-values calculated using two-sided Fisher's exact tests). **c,** Box plots (bands show the mean, each box extends between 1st and 3rd quartile, and whiskers extend 1.5x the interquartile range) showing mean expression of neoloop-associated genes from panel a in GSCs with neoloops (green) or astrocytes (black) with and without filtering of loci with >1.5 CNV. *: *P*=4.691e50 (without filtering) and 3.181e-12 (with filtering), two-sided Wilcoxon rank-sum test. Source data for this panel are provided as a Source Data file. **d,** Exemplary Hi-C contact maps from G412R around a 150-kbp deletion in the *ADAM9* locus. All neoloops forming across the breakpoint are indicated (blue circles). **e,** Box plots (drawn as in panel c) showing *ADAM9* expression in GSCs carrying or not neoloops in the locus. *: *P*=0.021, two-sided Student's t-test. Source data for this panel are provided as a Source Data file. **f,** Box plots (drawn as in panel c) showing *ADAM9* expression in TCGA GBM tumor and normal tissue data. *: *P*<0.01, two-sided Mann-Whitney U-test. **g,** Violin plots (medians indicated by white circles, and $1^{st}/3^{rd}$ quartile span by black boxes) showing no copy number variation in the *ADAM9* locus from TCGA GBM tumors with high (top 50%, magenta) or low *ADAM9* expression (bottom 50%, green). *P*=0.1, two-sided Mann-Whitney U-test. Source data for this panel are provided as a Source Data file. **h,** Kaplan-Meier disease-free survival analysis of TCGA GBM patients with *ADAM9* high and low expression. *P*-values were calculated using a two-sided log-rank test.

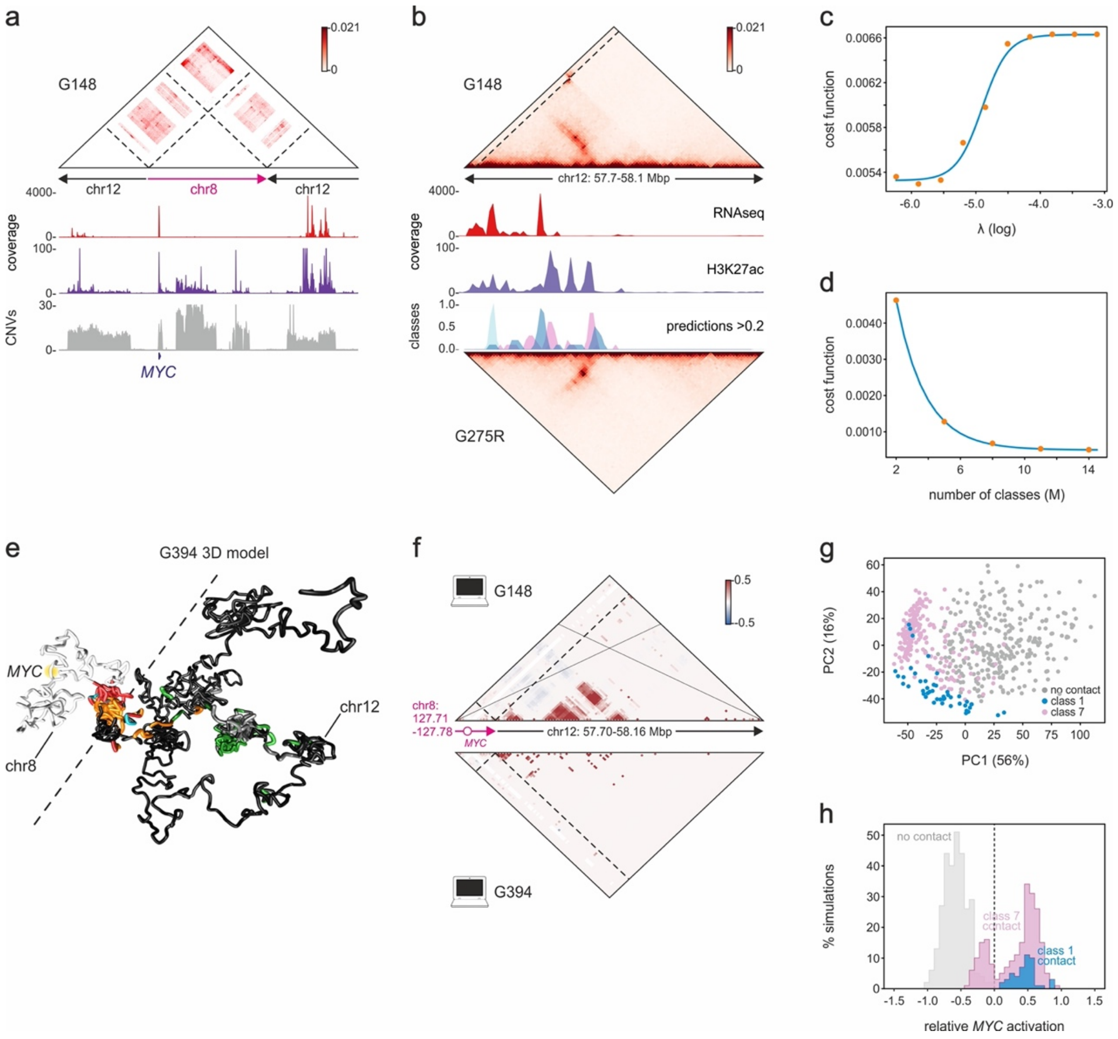

Supplementary Fig. 7. Characterization of a G148-specific translocation. a, Hi-C interactions connecting focally-amplified regions on chr8 and 12 into composite ecDNA. **b,** Hi-C contact map of the 1-Mbp chr12 segment of G148 translocated onto chr8 (dashed line: breakpoint) aligned to classes of polymer beads deduced from simulations in the same G275R region (bottom) using H3K27ac (middle) and RNA-seq signal (top). **c,** Plot showing the sigmoidal behavior of the cost function with varying regularization constants (λ) that penalize the abundance of binding sites during annealing optimization. **d,** As in panel b, but showing exponential decay of the cost function with increasing number of binding site classes (M). **e,** 3D rendering of a chr8 (white)-chr12 (black) translocation including *MYC* (yellow halo). Beads from classes that best predict folding are colored (green, red, and orange). **f,** *Top*: Triplet correlation coefficient of *MYC* with all pairs in the simulated G148 translocation. RNAseq- and H3K27ac-enriched regions form simultaneous contacts with *MYC* (positive correlation), but these occur rather independently from one another (negative correlation). *Bottom*: As above, but for G394 where no contacts form. **g,** PCA clustering of individual simulated *MYC* distance profiles (*N*=560) in G148 stratified by the degree of expression and by whether this is due to contacts with beads of enriched H2K27ac (blue), RNA-seq (pink) or to lack thereof (grey). **h,** Percent of simulated models plotted relative to the extrapolated mean *MYC* activation due to contacts with beads of H3K27ac (blue), RNA-seq (pink) or to lack thereof (grey).

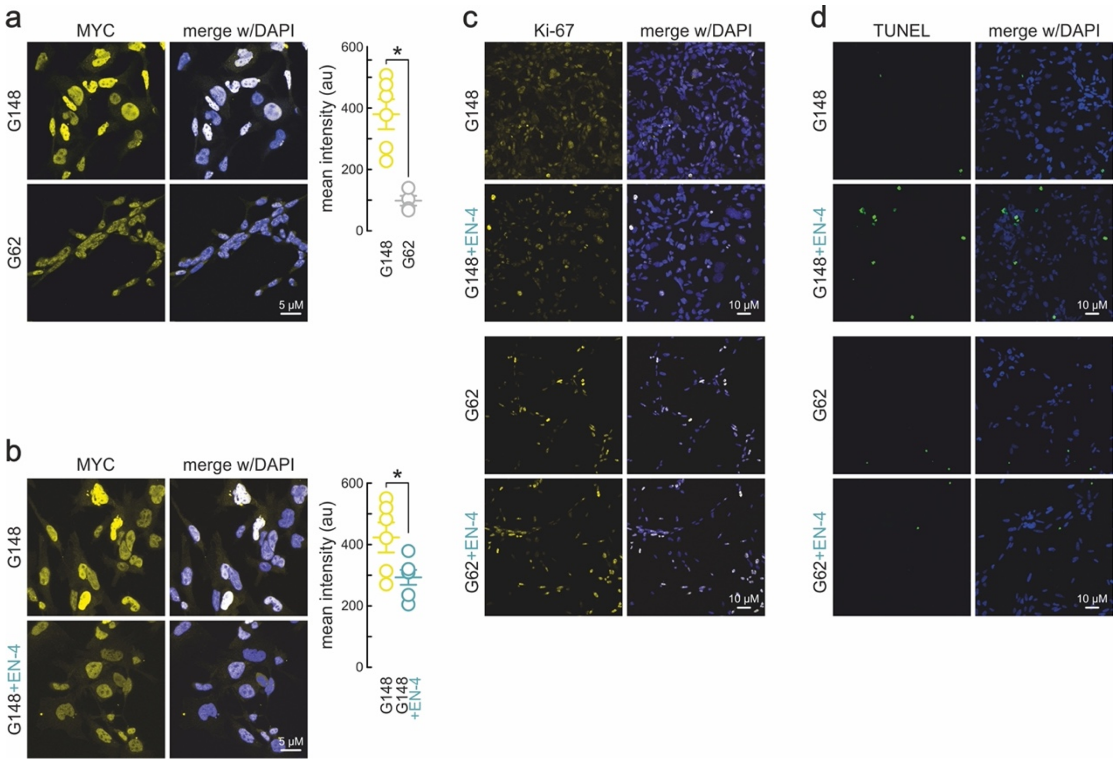

Supplementary Fig. 8. Selective inhibition of growth in MYC-overexpressing GSCs by EN-4. **a,** Left: Representative immunostainings from at least four independent experiments of G148 and G62 cells tested for MYC. Right: Plots showing mean MYC levels ±SD in each GSC line. **P*=0.00053, unpaired two-sided Student's t-test. Source data for this panel are provided as a Source Data file. **b,** As in panel a, but for G148 stained for MYC after treatment or not with 50 µM EN-4 for 48 h. **P*=0.0304, unpaired two-sided Student's t-test. Source data for this panel are provided as a Source Data file. **c,** As in panel a, but stained for Ki-67 after treatment or not with 50 µM EN-4 for 48 h. **d,** As in panel a, but TUNEL-stained after treatment or not with 50 µM EN-4 for 48 h.

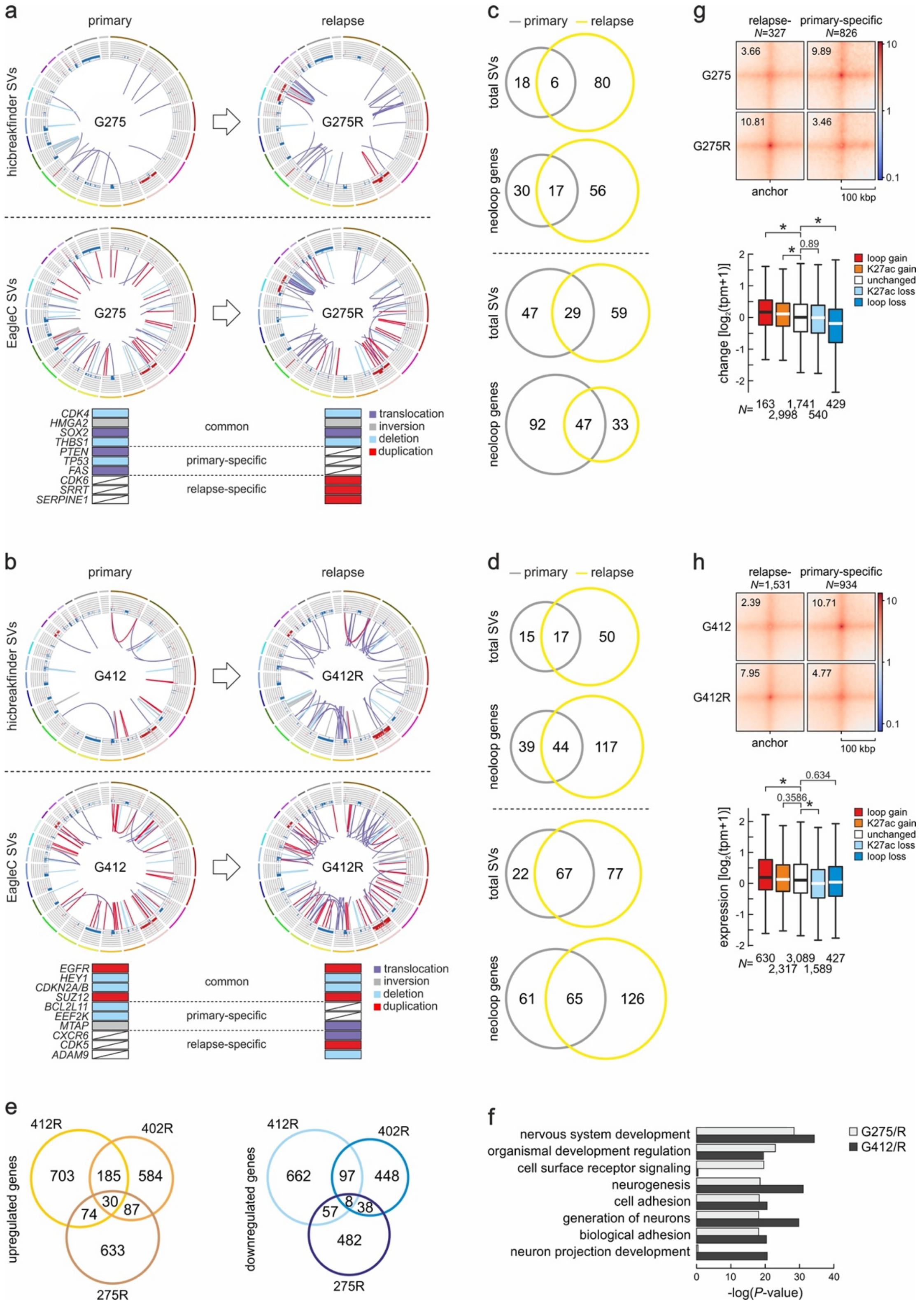

Supplementary Fig. 9. Comparison of SVs in primary versus relapse tumor GSCs. a, Circos plots of SVs and CNVs identified using *hicbreakfinder* (top) or EagleC (bottom) in the G275/275R primary-relapse pair. Outer tracks represent chromosomes, inner tracks indicate gain (red: >2 copies) or loss of genomic segments (blue: <2 copies), while lines depict inversions (grey), deletions (light blue), translocations (purple) or duplications (red). Aligned below the EagleC-

derived Circos plots are the top GBM-associated genes (gda>0.01) that are common to both lines or specific to each, and linked to a particular SV type (color-coded). **b,** As in panel a, but the G412/412R primary-relapse pair. **c,** Venn diagrams showing shared and unique SVs (top) or neoloop-associated genes (below) in primary (grey) and relapse G275/275R Hi-C data (yellow) deduced from *hicbreakfinder* (top) or EagleC analysis (bottom). **d,** As in panel c, but for the G412/412R primary-relapse pair. **e,** Venn diagrams showing shared and unique up- (left) and downregulated genes (right) from all three primary-relapse GSC pairs. **f,** GO terms associated with genes differentially-expressed in the primary versus relapse GSCs; *P*-values calculated using two-tailed Fisher's exact tests without multiple comparison adjustment. **g,** Left: APA plot for all loops specific to the G275 (primary) or G275R (relapse). Right: Box plots (bands show the mean, each box extends between 1st and 3rd quartile, and whiskers extend 1.5x the interquartile range) showing changes in the expression of genes associated with loops gained (red) or lost (blue), having increased (orange) or decreased H3K27ac (light blue), or not changing upon relapse (white). *: *P* = 5.623e-03 (loop gain vs unchanged), 4.719e-07 (K27ac gain vs unchanged) and 4.797e-08 (unchanged vs loop loss), two- sided Mann-Whitney U-test. Source data for this panel are provided as a Source Data file. **h,** As in panel g, but for the G412/R pair. *: *P* = 3.547e-04 (loop gain vs unchanged) and 8.602e-08 (unchanged vs K27ac loss), two- sided Mann-Whitney U-test. Source data for this panel are provided as a Source Data file.

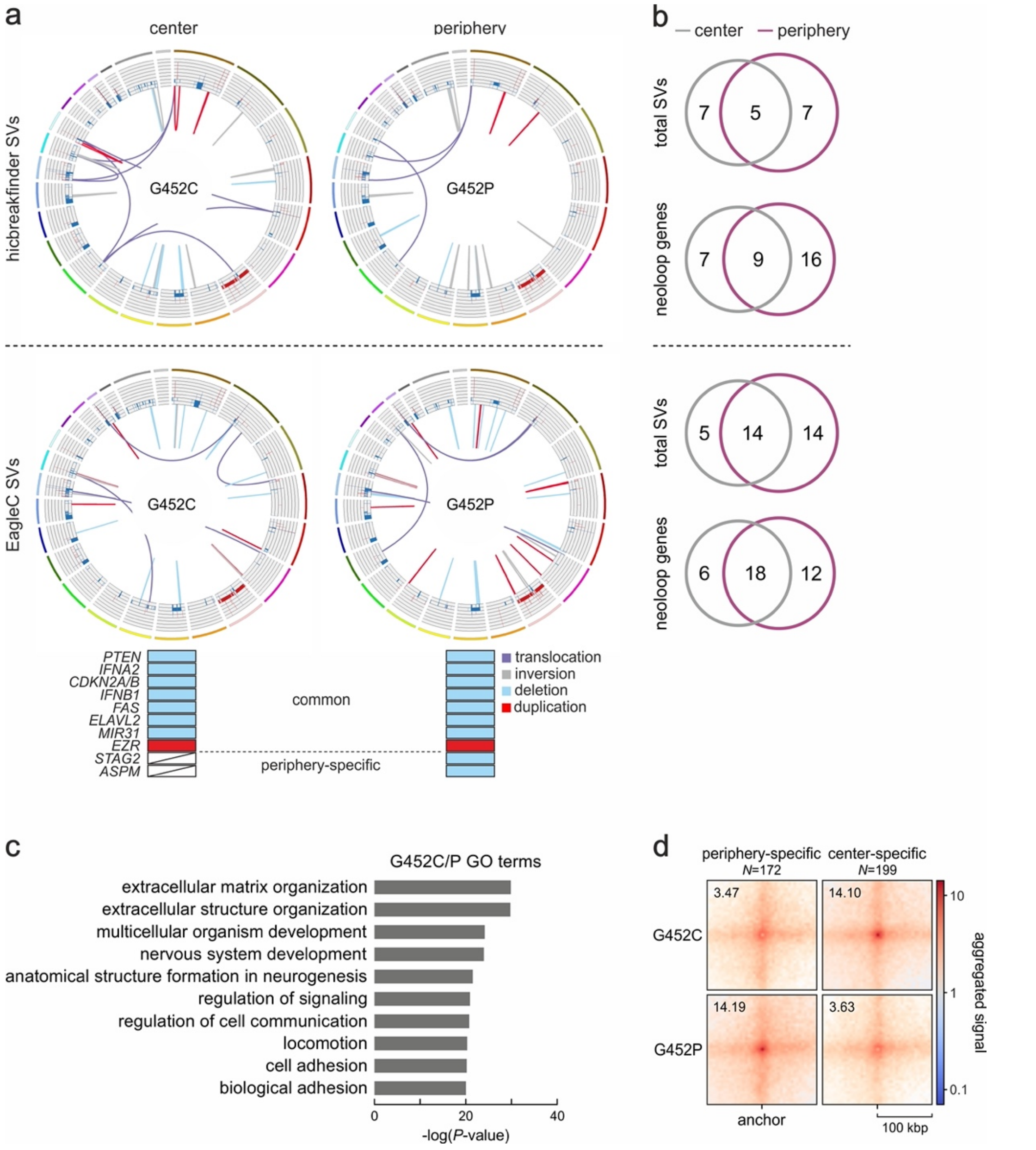

Supplementary Fig. 10. Comparison of SVs in the central versus peripheral part of a GBM tumor. a, Circos plots of SVs and CNVs in the G452C/P pair originating from the central and peripheral part of a single GBM tumor identified using *hicbreakfinder* (top) or EagleC (bottom). Outer tracks represent chromosomes, inner tracks indicate gain (red: >2 copies) or loss of genomic segments (blue: <2 copies), and lines depict deletions (light blue), inversions (grey), duplications (red) or translocations (purple). Aligned below each Circos plot are the top GBM-associated genes (gda>0.01) that are common to both lines or specific to G452P and linked to a particular SV type (color-coded). **b,** Venn diagrams showing shared and unique SVs (top) or neoloop-associated genes (bottom) in G452P/C data deduced from *hicbreakfinder* (top) or EagleC analysis (bottom). **c,** GO terms associated with genes differentially-expressed in 452C versus 452P; *P*-values calculated using two-tailed Fisher's exact tests without multiple comparison adjustment. **d,** APA plots for all loops specific to G452C or G452P.

Supplementary Table 1. Clinical features of GBM patients and tumors.

GSC#	age	sex	sympt	tumor type	tumor location	stupp	MGMT	IDH	EGFR	VEGF	PFS	OS
1	40	M	2.5	primary	temporal	yes	M	wt	neg	hyper	6	12.5
23	77	M	2	primary	parietal	no	UM	wt	neg	hyper	1	2
28	72	M	1.5	primary	frontal	yes	M	wt	neg	hyper	6	11.5
61	59	M	2	primary	occipital	no	UM	wt	pos	normal	3	6
62	64	M	36	relapse	frontal	yes	M	wt	neg	hyper	10	14
83	52	M	0.5	primary	temporal	yes	UM	wt	pos	hyper	4	8
120	53	M	25	relapse	parietal	yes	UM	wt	neg	hyper	8	16.5
148	55	M	6	relapse	parietal	yes	UM	wt	neg	hyper	5	8
163	56	M	5	primary	parietal	no	UM	wt	neg	normal	1	2
171	74	M	13	relapse	frontal	yes	M	wt	pos	normal	10	17
181	64	F	15	relapse	occipital	yes	M	wt	pos	hyper	12	17
208	66	M	22	relapse	temporal	yes	UM	wt	neg	hyper	20	33
213	50	M	18	relapse	frontal	yes	M	wt	neg	hyper	9	10.5
275<br>275R	58	M	2	primary<br>relapse	occipital	yes	M	wt	neg	hyper	6	12
318	69	M	25	relapse	temporal	yes	M	wt	neg	N/A	21	38
323	51	F	19	relapse	parietal	yes	UM	wt	neg	hyper	4	28
351	52	F	1	primary	temporal	yes	M	wt	pos	normal	84	84
390	49	M	0.5	relapse	temporal	yes	M	wt	pos	hyper	23	32.5
394	64	M	1	relapse	frontal	yes	M	wt	pos	hyper	5	23
402<br>402R*	58	M	0.5	primary<br>relapse	parietal	yes	UM	wt	neg	hyper	9	23
412<br>412R**	56	F	1	primary<br>relapse	frontal	no	UM	wt	neg	hyper	22	31
450	76	F	1	primary	temporal	no	M	wt	neg	hyper	3	6
452P/C[§]	67	F	46	relapse	temporal	no	M	wt	pos	hyper	1.5	2
457	59	F	0.5	relapse	frontal	yes	M	wt	pos	hyper	9.5	14.5

Age is displayed in years; symptoms' duration is displayed in months; M/UM, methylated/unmethylated; N/A, not available; PFS, progression-free survival displayed in months; OS, overall survival displayed in months; *initially named 428; **initially named 486; [§]P/C, biopsy peripheral/central to the tumor.

Supplementary Table 2. Statistics and quality metrics of Hi-C experiments.

GSC#	total read pairs	% uniquely mapped	valid read pairs	% long-range interactions	% trans interactions
1	625,787,061	82.45%	397,322,201	38.17%	27.45%
23	896,831,218	74.97%	517,118,202	48.77%	23.18%
28	465,573,200	82.77%	292,536,305	32.87%	26.50%
61	469,087,274	76.98%	300,832,405	44.71%	21.66%
62	556,915,214	78.36%	363,717,617	50.87%	18.05%
83	640,920,736	77.47%	405,480,447	52.68%	17.51%
120	576,586,441	80.57%	375,550,401	45.03%	20.79%
148	566,598,758	78.58%	367,401,496	44.31%	24.01%
163	658,302,887	80.73%	414,448,463	41.55%	18.65%
171	633,593,298	80.15%	419,365,905	43.29%	20.96%
181	302,003,570	79.97%	196,344,108	41.91%	22.81%
208r1	660,900,454	83.97%	431,713,920	32.44%	16.05%
208r2	628,126,052	78.23%	386,221,051	33.85%	42.22%
213r1	874,108,698	85.06%	519,662,045	24.35%	21.84%
213r2	622,608,520	77.22%	387,808,004	43.20%	29.62%
275	694,194,997	79.27%	446,298,283	43.02%	20.32%
275R	506,647,616	79.57%	330,626,223	41.62%	20.36%
318	575,672,446	76.63%	368,523,495	47.61%	19.29%
323	605,260,377	78.10%	391,679,652	47.02%	22.11%
351	743,980,073	81.42%	490,730,702	54.00%	18.27%
390	619,497,564	83.23%	401,753,521	33.80%	24.30%
394	657,571,552	79.38%	422,381,344	47.95%	22.94%
402	623,636,610	78.58%	360,887,948	51.00%	23.18%
402R	727,935,611	81.14%	473,206,854	48.53%	25.29%
412	550,364,451	79.88%	363,954,866	47.25%	18.87%
412R	708,374,421	81.80%	452,528,651	50.61%	17.97%
450	750,273,787	77.33%	463,004,321	48.16%	19.66%
452C	653,145,500	78.91%	415,038,329	44.64%	21.29%
452P	597,888,199	79.03%	384,384,412	47.34%	21.86%
457	855,162,397	84.10%	487,186,793	30.07%	25.35%

r1/r2 designate independent replicates; R designates the relapse sample in a GSC pair.
