# Supplementary material for: Pervasive structural heterogeneity rewires glioblastoma chromosomes to sustain patient-specific transcriptional programs
Source: Nat Commun. 2024 May 9;15:3905. doi: 10.1038/s41467-024-48053-2 (PMC11082206; doi:10.1038/s41467-024-48053-2)
Supplement: Supplementary file 3 — Description of Additional Supplementary Files [file 41467_2024_48053_MOESM3_ESM.pdf]

Description of Additional Supplementary Files

File Name: Supplementary Data 1
Description: Supplementary Table 3. Catalogue of all CNVs, SVs, neoloops, and gene fusion eventsidentified using EagleC or hicbreakfinder to Hi-C data from all GSCs.

File Name: Supplementary Data 2
Description: Catalogue of all GBM-associated genes (gda>0.01) associated with CNVs and EagleC- or hicbreakfinder-deduced SVs in GSC sample pairs.
