# Supplementary material for: Pervasive structural heterogeneity rewires glioblastoma chromosomes to sustain patient-specific transcriptional programs
Source: Nat Commun. 2024 May 9;15:3905. doi: 10.1038/s41467-024-48053-2 (PMC11082206; doi:10.1038/s41467-024-48053-2)
Supplement: Supplementary file 6 — Reporting Summary [file 41467_2024_48053_MOESM6_ESM.pdf]

Corresponding author(s): Argyris Papantonis and Roberto Pallini

Last updated by author(s): Apr 5, 2024

Reporting Summary

Nature Portfolio wishes to improve the reproducibility of the work that we publish. This form provides structure for consistency and transparency in reporting. For further information on Nature Portfolio policies, see our Editorial Policies and the Editorial Policy Checklist.

Statistics

For all statistical analyses, confirm that the following items are present in the figure legend, table legend, main text, or Methods section.

n/a	Confirmed	
☐	☒	The exact sample size (*n*) for each experimental group/condition, given as a discrete number and unit of measurement
☐	☒	A statement on whether measurements were taken from distinct samples or whether the same sample was measured repeatedly
☐	☒	The statistical test(s) used AND whether they are one- or two-sided<br>*Only common tests should be described solely by name; describe more complex techniques in the Methods section.*
☒	☐	A description of all covariates tested
☐	☒	A description of any assumptions or corrections, such as tests of normality and adjustment for multiple comparisons
☐	☒	A full description of the statistical parameters including central tendency (e.g. means) or other basic estimates (e.g. regression coefficient) AND variation (e.g. standard deviation) or associated estimates of uncertainty (e.g. confidence intervals)
☐	☒	For null hypothesis testing, the test statistic (e.g. *F*, *t*, *r*) with confidence intervals, effect sizes, degrees of freedom and *P* value noted<br>*Give P values as exact values whenever suitable.*
☒	☐	For Bayesian analysis, information on the choice of priors and Markov chain Monte Carlo settings
☒	☐	For hierarchical and complex designs, identification of the appropriate level for tests and full reporting of outcomes
☐	☒	Estimates of effect sizes (e.g. Cohen's *d*, Pearson's *r*), indicating how they were calculated

Our web collection on statistics for biologists contains articles on many of the points above.

Software and code

Policy information about availability of computer code

Data collection

No software was used for data collection.

Data analysis

For basic analysis the following standard tools were used.
-bwa mem (v0.7.17;https://bio-bwa.sourceforge.net/)
-runHiC (v0.8.4-r1; https://zenodo.org/badge/doi/10.5281/zenodo)
-cooler (v0.8.6;https://cooler.readthedocs.io/en/latest/index.html)
-EagleC (v0.1.3;https://github.com/XiaoTaoWang/EagleC)
-Neoloopfinder (v0.3.0.post4;https://github.com/XiaoTaoWang/NeoLoopFinder)
-HiCrep (v0.2.3;https://github.com/TaoYang-dev/hicrep)
-cooltools (v0.3.2;https://cooltools.readthedocs.io/en/latest/#)
-peakachu (v1.2.0;https://github.com/open2c/cooltools)
-STAR (v2.6.0c;https://github.com/alexdobin/STAR)
-RSEM (v1.3.3;https://github.com/deweylab/RSEM)
-deepTools2 (v3.5.1;https://github.com/deeptools/deepTools)
-Bowtie2 (v2.3.4.1;https://bowtie-bio.sourceforge.net/bowtie2/manual.shtml)
-cBioPortal (v3.5.3;https://www.cbioportal.org/)
-Picard tools (v2.20.7;https://broadinstitute.github.io/picard/)
-SEACR (v1.3;https://github.com/FredHutch/SEACR)
-CNVkit (v0.9.9;https://github.com/etal/cnvkit)
-GEPIA2 (v7.0;http://gepia2.cancer-pku.cn/)
-DisGenet Database (v7.0;https://www.disgenet.org/)

-Arriba (v2.3.0;https://github.com/suhrig/arriba)
Custom script used to analyse Hi-C, RNA-seq, WGS and CUT&Tag data is available at https://github.com/xieting0603/GBM; the custom code used to perform simulations is available at https://github.com/marianoimperatore/MeanFieldChromatin.git.

For manuscripts utilizing custom algorithms or software that are central to the research but not yet described in published literature, software must be made available to editors and reviewers. We strongly encourage code deposition in a community repository (e.g. GitHub). See the Nature Portfolio guidelines for submitting code & software for further information.

Data

Policy information about availability of data

All manuscripts must include a data availability statement. This statement should provide the following information, where applicable:

- Accession codes, unique identifiers, or web links for publicly available datasets
- A description of any restrictions on data availability
- For clinical datasets or third party data, please ensure that the statement adheres to our policy

Due to national patient protection policy, raw NGS data can only be released upon request and ethics approval. Minimally processed data (i.e., following mapping to the reference human genome) that do not contain identifiable information can be freely accessed via the NCBI Gene Expression Omnibus (GEO) under accession number GSE229966 (https://www.ncbi.nlm.nih.gov/geo/query/acc.cgi?acc=GSE229966).

Research involving human participants, their data, or biological material

Policy information about studies with human participants or human data. See also policy information about sex, gender (identity/presentation), and sexual orientation and race, ethnicity and racism.

Reporting on sex and gender	Patient recruitment and study strategy was indiscriminatory towards biological sex/gender, which is anyway reported in Supplementary Table 1. Note that sex/gender was self-reported. We do not present data disaggregated for sex and gender for most analyses (SV detection is presented on a patient-by-patient basis in Supplementary Fig. 2) as the study design and patient numbers would not be sufficient for this (only 7 female GBM patients could be recruited out of a total of 24), and post-hoc analyses are discouraged.
Reporting on race, ethnicity, or other socially relevant groupings	n/a
Population characteristics	n/a
Recruitment	Recruitment was voluntary for diagnosed GBM patients following informed consent, and is described in the relevant Methods section.
Ethics oversight	Research reported here complies with all relevant national and international regulations, including the Declaration of Helsinki. The collection and processing of all samples was approved by the Ethics Board of the University Hospital, Catholic University of Rome (Prot. ID CE 2253) with informed consent obtained from all GBM patients.

Note that full information on the approval of the study protocol must also be provided in the manuscript.

Field-specific reporting

Please select the one below that is the best fit for your research. If you are not sure, read the appropriate sections before making your selection.

☒ Life sciences ☐ Behavioural & social sciences ☐ Ecological, evolutionary & environmental sciences

For a reference copy of the document with all sections, see nature.com/documents/nr-reporting-summary-flat.pdf

Life sciences study design

All studies must disclose on these points even when the disclosure is negative.

Sample size	We compiled a cohort of 28 patient-derived glioblastoma stem cell-like lines, including three primary-relapse GSC pairs. We try to include both primary and relapse samples and as much as primary-relapse pairs. The sample size is restrained by the generally limited accessibility to the patient samples. No sample size calculation was performed.
Data exclusions	No data was excluded.
Replication	Two biological replicates were applied to two randomly-selected lines. All attempts at replication were consistently successful.
Randomization	This is not relevant to our study.

Blinding	This is not relevant to our study.

Reporting for specific materials, systems and methods

We require information from authors about some types of materials, experimental systems and methods used in many studies. Here, indicate whether each material, system or method listed is relevant to your study. If you are not sure if a list item applies to your research, read the appropriate section before selecting a response.

Materials & experimental systems

n/a	Involved in the study
☐	☒ Antibodies
☐	☒ Eukaryotic cell lines
☒	☐ Palaeontology and archaeology
☒	☐ Animals and other organisms
☒	☐ Clinical data
☒	☐ Dual use research of concern
☒	☐ Plants

Methods

n/a	Involved in the study
☒	☐ ChIP-seq
☒	☐ Flow cytometry
☒	☐ MRI-based neuroimaging

Antibodies

Antibodies used	a-CTCF (Active Motif #61311; 1 µg per IP), a-H3K27ac (Active Motif #9133; 1 µg per IP), a-Ki67 (Merck Millipore, AB9260; 1:1000 dilution), a-MYC (Proteintech, #10828-1-AP; 1:1000 dilution)
Validation	All antibodies used in this study are commercially validated via western blots against protein extracts from human cells.

Eukaryotic cell lines

Policy information about cell lines and Sex and Gender in Research

Cell line source(s)	Glioblastoma tumors from 24 patients who underwent surgery at diagnosis (n=11) or relapse (n=17, as 3 initially-resected patients were also part of the relapse group) at the Institute of Neurosurgery, Catholic University of Rome, were used to produce 28 glioblastoma stem-like cell lines.
Authentication	n/a
Mycoplasma contamination	We confirm that our GSC lines were regularly tested and do not have mycoplasma contamination.
Commonly misidentified lines (See ICLAC register)	We confirm that none of the patient-derived lines we used are known misidentified lines
